# Quantum Dots Mediated Heterojunction Coupling MoSe_2_ Photoanode for Photoelectrochemical Water Splitting

**DOI:** 10.3390/molecules29051070

**Published:** 2024-02-29

**Authors:** Lin Zhang, Jiana Sun, Mengmeng Zhao, Yuxuan Wei, Taigang Luo, Zhengping Zhao, Yibo Yan

**Affiliations:** 1 Frontiers Science Center for Flexible Electronics (FSCFE), Xi’an Institute of Flexible Electronics (IFE) and Xi’an Institute of Biomedical Materials & Engineering (IBME), Northwestern Polytechnical University, 127 West Youyi Road, Xi’an 710072, China; 2 Zhijiang College, Zhejiang University of Technology, Hangzhou 310014, China

**Keywords:** photoelectrochemical, hydrogen evolution, heterojunction, molybdenum selenide, quantum dot, photoanode, heteroatom doping

## Abstract

Graphene quantum dots (GQDs) possess the photosensitive absorption for photoelectrochemical hydrogen evolution owing to special band structures, whereas they usually confront with photo-corrosion or undesired charge recombination during photoelectrochemical reactions. Hence, we establish the heterojunction between GQDs and MoSe_2_ sheets via a hydrothermal process for improved stability and performance. Photoanodic water splitting with hydrogen evolution boosted by the heteroatom doped N,S-GQDs/MoSe_2_ heterojunction has been attained due to the abundant active sites, promoted charge separation and transfer kinetics with reduced energy barriers. Diphasic 1T and 2H MoSe_2_ sheet-hybridized quantum dots contribute to the Schottky heterojunction, which can play a key role in expedited carrier transport to inhibit accumulative photo-corrosion and increase photocurrent. Heteroatom dopants lead to favored energy band matching, bandgap narrowing, stronger light absorption and high photocurrent density. The external quantum efficiency of the doped heterojunction has been elevated twofold over that of the non-doped pristine heterojunction. Modification of the graphene quantum dots and MoSe_2_ heterojunction demonstrate a viable and adaptable platform toward photoelectrochemical hydrogen evolution processes.

## 1. Introduction

Hydrogen evolution via a photoelectrochemical (PEC) system has attracted numerous curiosities for sustainable clean-energy transformation. The PEC catalysis often demands the appropriate electronic energy levels, bandgap structures and band edge positions. A series of metal oxides (e.g., TiO_2_) were investigated as light harvesters to absorb photons [1,2]. Nonetheless, the majority of metal oxides can only absorb partial simulated sunlight or visible light due to the large bandgap and short exciton lifetime due to the fast recombination of photogenerated carriers. For comparison, metal sulfides and nitrides have exhibited better electrical properties and a more suitable bandgap structure for absorbing more visible light than metal oxides [3,4,5,6,7]. Additionally, graphene quantum dots (GQDs) have emerged as one of the hottest light harvesters with abundant active sites and a bandgap suitable for photocatalytic and PEC applications [8,9], except for the inevitable charge recombination and photo-corrosion phenomenon. After coupling with appropriate cocatalysts, GQDs could largely promote the overall catalytic performance.

For improvement of the photostability, photocatalytic and photoelectrochemical activities of GQDs, surface modifications, such as grafting electron-donating functional groups and conjugating polyaromatic groups to enlarge the π conjugation system, have been employed in electronic energy band engineering for hydrogen evolution and CO_2_ reduction [10]. An intramolecular Z-scheme existed, consisting of p-type and n-type domains induced by electron-withdrawing and electron-donating species, respectively, along with the sp^2^-carbon ohmic contact in between. The Z-scheme configuration assisted to couple redox reactions, averting the recombination of photogenerated electron-hole pairs. However, the yield of organic synthesis was complicated with side products. Facile synthesis was demanded with feasible and scalable operation processes. Heterojunctions between GQDs and various materials, such as graphene, metal oxides and sulfides, have been employed to improve photogenerated charge separation and inhibit recombination for elevated photoelectrochemical performance. GQDs and a boron-hybridized g-C_3_N_4_ heterojunction possessed an ultrahigh specific surface area, enriched active sites, strong photoelectric activity and facilitated charge transfer ability [11]. Photocurrent was significantly enhanced by introduction of GQDs and boron in a hybridized heterojunction compared to that of pure g-C_3_N_4_ nanosheets. Furthermore, the photocurrent linearly increased as dopamine concentration increased, showing high selectivity and stability for PEC detection of dopamine. In addition, GQDs could serve as the intercalation agent to exfoliate 2D materials such as graphene and form a 0D/2D van der Waals heterojunction for PEC water splitting [8]. Heteroatom doping modified the bandgap structure and red-shifted the photoluminescence (PL) and UV–vis light absorption wavelength spectrum for improved photogenerated charge separation, carrier transfer, PEC catalytic activity and reduced Schottky barrier on the interface of the heterojunction.

Molybdenum selenide (MoSe_2_) is a typical transition-metal dichalcogenide (TMD) material and frequently studied for the hydrogen evolution reaction (HER) [12,13]. Due to the favorable hydrogen adsorption energy, the MoSe_2_ defective sites are taken as the catalytic active sites [14,15]. Subsequently, many studies have been focused on the surface engineering of defects and active sites for enhancement of catalytic performance [16,17,18]. In photoelectrochemical catalytic HER, semiconductive 2H phase MoSe_2_ efficiently improved the charge separation of photogenerated electron-hole pairs and stability [19,20]. However, the photogenerated charge separation and transfer were usually limited on the surface areas. To increase the catalytic effective surface area and exciton lifetime, facilitate photogenerated carrier separation and retard charge recombination, hybridization of MoSe_2_ with other materials was assembled to form heterojunctions to improve overall performance [21,22,23]. A micro/nanoflower heterojunction between MoSe_2_ and g-C_3_N_4_ nanosheets facilitated the PEC faradaic efficiency and production yield of NH_3_ formed by N_2_. The elevated PEC performance was due to the hierarchical architecture, large effective surface area, abundant active sites, enhanced light absorption and improved charge separation by the hierarchical heterojunction structure [24]. Moreover, a Z-scheme heterojunction photoanode of MoSe_2_ nanosheets conjugated CdS-ZnO arrays exhibited high incident-photon-to-current efficiency and enabled the photoanode, achieving high performance PEC hydrogen generation. The cooperation between a tunable Z-scheme heterojunction with double internal electric fields and construction of a 3D cross-linked network structure opened up a promising prospect for designing high-performance PEC water splitting devices [25]. In addition, partial conductivity also played a key role in the determination of PEC catalytic performance. It was reported that a core-shell heterostructure of (1T-2H) MoSe_2_ on TiO_2_ nanorods provided an S-scheme heterojunction for charge separation and transfer in PEC catalytic activities for H_2_O_2_ production, where MoSe_2_ could suppress the PEC decomposition of H_2_O_2_ by decreasing its adsorption by the photoanode surface. 1T MoSe_2_ was quasi-conductive, serving as the bridge between semiconductive 2H MoSe_2_ and TiO_2_ [26]. The 1T phase of MoSe_2_ reduced the resistance/barriers and boosted charge transport kinetics, inducing dynamic improvement in PEC performance.

## 2. Results

### 2.1. Synthesis and Characterization

#### 2.1.1. Preparation Process

Hydrothermal synthesis of N,S-GQDs was implemented by using citric acid as the carbon source, urea as the nitrogen source and thiourea as the sulfur source. A total of 1 mmol citric acid, 3 mmol urea and 3 mmol thiourea were dissolved in 5 mL of deionized water and sonicated for 10 min to dissolve uniformly and form a clear solution. Then, the solution was transferred to a 20 mL high-pressure autoclave and heated in an oven at 160 °C for 4 h. After the reaction was completed, it naturally cooled to room temperature to undergo continuous dialysis and freeze drying; the targeted product achieved N,S-GQDs. For comparison, pristine GQDs were prepared similarly without using urea or thiourea as the N or S sources, respectively. Afterwards, a quantum dot mediated MoSe_2_ heterojunction was synthesized by another hydrothermal process of two mixed solutions: 4 mmol Se powder dispersed in 10 mL N_2_H_4_·H_2_O and 0.353 g (NH_4_)_6_Mo_7_O_24_·4H_2_O and 5 mg N,S-GQDs dissolved in 20 mL H_2_O. The hydrothermal condition was set at 150 °C for 48 h to convert Mo_7_O_24_^6−^ into Mo^4+^ for the MoSe_2_ generation. The resulting sample was rinsed and centrifugated six times to obtain the target heterojunction samples.

#### 2.1.2. Morphology Characterizations

**Figure 1:**
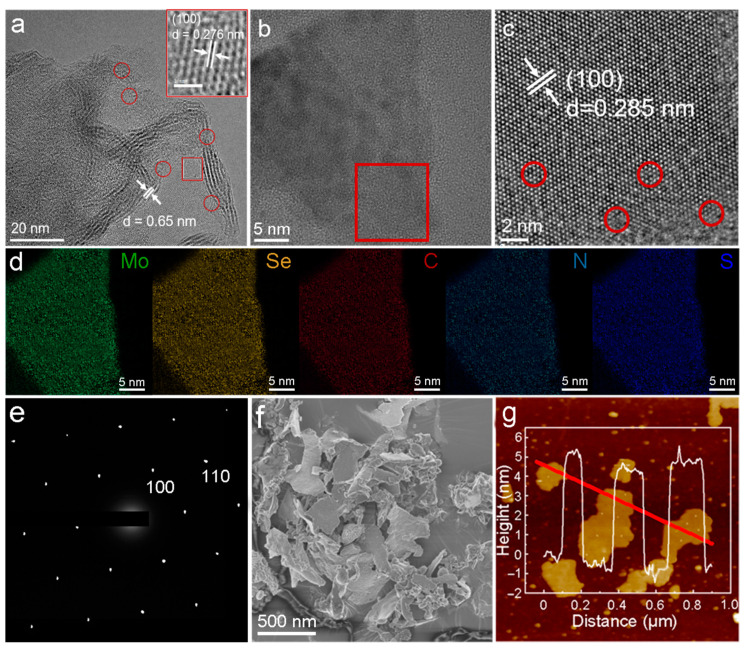
Structure characterizations (**a**) TEM, (**b**) HRTEM, (**c**) magnified HRTEM of red box area in (**b**), (**d**) EDS mapping, (**e**) SAED, (**f**) SEM and (**g**) AFM of N,S-GQDs/MoSe_2_.

In Figure 1a, the transmission electron microscope (TEM) image of the MoSe_2_ nanosheets demonstrates that the stacked nanosheets consist of approximately three to eight monolayers. And the interlayer spacing is approximately 0.65 nm, corresponding to the interlayer spacing of 2H MoSe_2_, indicating the existence of the 2H phase MoSe_2_ [23].Interestingly, a triangular lattice structure (octahedral coordination) has been magnified and highlighted in the red box with a lattice spacing of approximately 0.276 nm, which is close to the 1T phase (100) crystal plane spacing of MoSe_2_ [22]. These results substantiated the existence of the 1T phase and suggested the coexistence of the 1T and 2H phases, where the 2H phase is thermodynamically stable and the 1T phase is much more conductive with a higher electron density, which has been caused by the highly reductive N_2_H_4_ agent. In addition, there are numerous defects on the basal plane and edge sites of MoSe_2_, as indicated by the red circles, which may be ascribed to the lattice mismatching, resulting in a large number of internal and edge defects and contributing to increased active sites. The high-resolution transmission electron microscope (HRTEM) of N,S-GQDs on the MoSe_2_ nanosheets is shown in Figure 1b. Quantum dots smaller than 5 nm were evenly dispersed on the surface of the MoSe_2_ nanosheets. The magnified HRTEM of the red box area is exhibited in Figure 1c, showing a periodic lattice arrangement with a lattice spacing of 0.285 nm, indexed to the 2H phase (100) crystal plane spacing of MoSe_2_. As indicated by the red circles, there are abundant defective vacancies, lattice mismatching and lattice disorders at the edge sites, which may increase the active sites and active surface areas. Energy dispersive spectroscopy (EDS) characterized elemental mapping is shown in Figure 1d. The element distribution of C, N and S is mostly the same as that of Mo and Se, and the distribution is relatively uniform, which proves that N,S-GQDs are evenly dispersed on the MoSe_2_ support. The selected area electron diffraction (SAED) pattern (Figure 1e) shows a clear and highly symmetrical hexagonal or triangular pattern, implying that the grown MoSe_2_ nanosheets have hexagonal/triangular crystal structures. Scan electron microscopy (SEM) observation (Figure 1f) exhibited the relatively uniform size of the nanosheets without obvious reaggregation toward large sheets. There are some curls on the edges due to their relatively thin thickness and the decreased stiffness. To determine the thickness and number of atomic layers, the atomic force microscopy (AFM) analysis was performed (Figure 1g) [27,28]. The AFM results, consistent with the SEM and TEM results, demonstrate large layers and small fragments, showing quantum dots stacked on the MoSe_2_ layers. The layer thickness ranged from 4.6 to 5.4 nm. And the number of layers approximately ranged from six to eight, calculated based on the monolayer thickness of approximately 0.65 nm and the additional distance induced by the influence of interatomic repulsion, capillary and adhesion forces.

#### 2.1.3. Properties Characterizations

**Figure 2:**
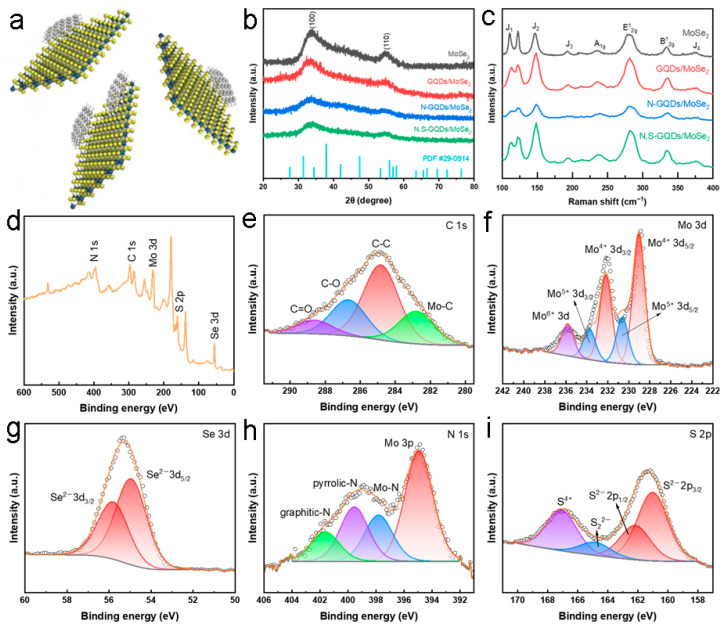
(**a**) Scheme of the heterojunction structure. (**b**) XRD, (**c**) RAMAN, (**d**) overall XPS, (**e**) C 1s, (**f**) Mo 3d, (**g**) Se 3d, (**h**) N 1s and (**i**) S 2p high resolution XPS patterns.

The heterojunction structure of N,S-GQDs/MoSe_2_ is illustrated in Figure 2a. X-ray diffraction (XRD) spectra (Figure 2b) show two main diffraction peaks at 33.7° and 55°, significantly differentiated from the standard PDF card of 2H phase MoSe_2_ (JCPDs NO. 29-0914). The two peaks at 33.7° and 55° are indexed to the (100) and (110) crystal planes, respectively. The small lattice spacing of 0.276 nm of 1T MoSe_2_ corresponds to the (100) crystal plane peak (33.7°), according to the Bragg law [29]. The Raman spectra in the range of 100–400 cm^−1^ are displayed in Figure 2c. The out-of-plane and in-plane A_1g_ and E^1^_2g_ vibration modes were observed at approximately 237 cm^−1^ and 281 cm^−1^, respectively, attributed to the semiconductive 2H phase structure of MoSe_2_. There is an in-plane B^1^_2g_ vibration mode at approximately 335 cm^−1^, attributed to the translational symmetry disruption occurring in the few-layer MoSe_2_ nanosheets [30]. In addition, there are some new Raman peaks located at 110 cm^−1^, 148 cm^−1^, 192 cm^−1^ and 375 cm^−1^, labeled as J_1_, J_2_, J_3_ and J_4_ vibrational modes, respectively, attributed to the metal 1T phase of MoSe_2_. The vibration modes of J_1_, J_2_ and J_3_ are consistent with the Raman peaks of 1T-MoSe_2_ prepared by Miao et al. [31], indicating good formation of the 1T phase MoSe_2_ nanosheets. Meanwhile, the J_4_ mode has also been demonstrated in the report by Saghar et al. [32]. Based on the analysis of the Raman peak positions, it is further demonstrated that the hydrothermal synthesized MoSe_2_ has a dual-phase mixed structure of 1T and 2H.

Figure 2d shows the overall XPS spectra of N,S-GQDs/MoSe_2_, showing the coexistence of C, Mo, Se, N and S elements, which is consistent with the results of EDS mapping. Figure 2d–i show the high-resolution XPS spectra of C 1s, Mo 3d, Se 3d, N 1s and S 2p, respectively. The characteristic peaks located at 282.8 eV, 284.8 eV, 286.7 eV and 288.6 eV in the C 1s XPS spectra are indexed to Mo-C, C-C, C-O and C=O, respectively (Figure 2e). The presence of Mo-C bonds indicates that, during hydrothermal reactions, N,S-GQDs form a certain number of covalent bonds with Mo^4+^, contributing to the conductivity and active sites of the heterojunction. There are five characteristic peaks in the Mo 3d XPS spectra (Figure 2f), among which the characteristic peaks at binding energies of 229.1 eV, 232.3 eV, 230.5 eV, 233.7 eV and 235.8 eV correspond to Mo^4+^ 3d_5/2_, Mo^4+^ 3d_3/2_, Mo^5+^ 3d_5/2_, Mo^5+^ 3d_3/2_ and Mo^6+^ 3d, respectively [33]. The presence of Mo^5+^ 3d_5/2_, Mo^5+^ 3d_3/2_ and Mo^6+^ 3d may be related to the incomplete reduction of the Mo precursor (NH_4_)_6_Mo_7_O_24_·4H_2_O. Interestingly, the binding energies of Mo^4+^ 3d_5/2_ and Mo^4+^ 3d_3/2_ shifted by ~0.9 eV compared to the standard binding energies of 2H MoSe_2_ (Mo^4+^ 3d_5/2_ and Mo^4+^ 3d_3/2_ at 228.2 eV and 231.4 eV, respectively), implying that 1T MoSe_2_ can engender from the hydrothermal method [34]. In the Se 3d XPS spectra (Figure 2g), the characteristic peaks at 54.8 eV and 55.7 eV belong to Se^2-^ 3d_5/2_ and Se^2-^ 3d_3/2_, respectively, implying the −2 valence state of Se [35]. The N 1s XPS (Figure 2h) can be deconvoluted into four characteristic peaks at binding energies of 395 eV, 397.8 eV, 399.5 eV and 401.5 eV indexed to the Mo 3p, Mo-N, pyrrolic-N and graphitic-N, respectively. The presence of Mo-N indicates that N,S-GQDs and MoSe_2_ are not merely physically mixed but also form Mo-N coordination bonds [36]. Graphene-N has been formed via substituting C atoms by N atoms. The S 2p XPS (Figure 2i) can be deconvoluted into four characteristic peaks, and the main S^2−^ 2p_3/2_ and S^2−^ 2p_1/2_ peaks are localized at 161 eV and 162.2 eV, respectively [37]. The characteristic peak at 164.8 eV indexed to the bridged disulfide S_2_^2−^, while the peak at 167.1 eV may be attributed to S^4+^, caused by the incomplete oxidation of S^2−^.

### 2.2. PEC Studies

#### 2.2.1. PEC HER Performance

**Figure 3:**
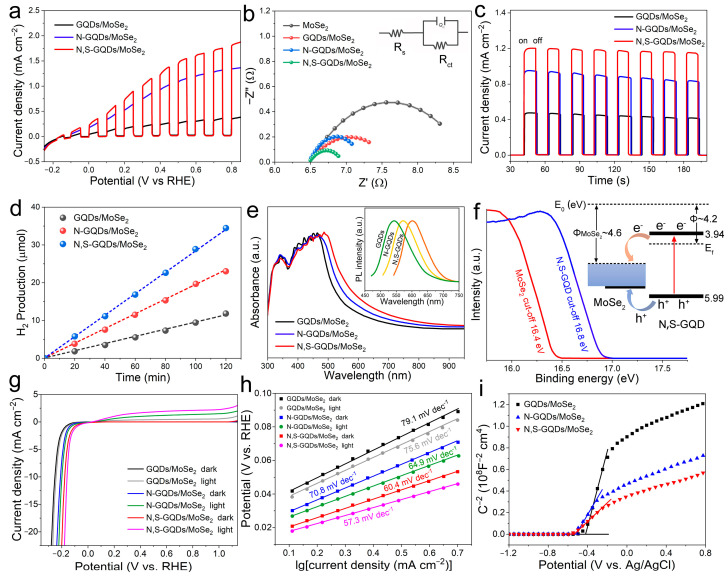
(**a**) Chopped current density–voltage measurements. (**b**) Nyquist impedance spectroscopy of different heterojunctions in the frequency range of 5 MHz to 10 Hz at a potential of 0 V vs. RHE. (**c**) Photo-response measurement at applied voltage of 0.4 V in 10% lactic acid. (**d**) Photocatalytic H_2_ generation. (**e**) UV–visible absorption spectra. Inset shows the photoluminescence of distinct quantum dots. (**f**) UPS spectra with inset for energy band alignment diagram. (**g**) LSV of HER polarization curves of different samples with or without light irradiation. (**h**) Tafel slopes of the LSV polarization tests for different samples with or without light irradiation. (**i**) The Mott-Schottky plots of the GQDs/MoSe_2_, N-GQDs/MoSe_2_ and N,S-GQDs/MoSe_2_ heterojunctions.

The PEC performance was measured with a three-electrode system using 10%(*v*/*v*) lactic acid as the electrolyte (Ph = 3). Linear sweep voltammetry (LSV) was conducted to obtain polarized curves during the fourth cycle. Light on/off irradiation was applied periodically chopped 1 sun (AM 1.5) at the intensity of 100 mW cm^–2^, and the photocurrent density was tested as the dependent variable of potential (Figure 3a). Negligible light-off currents indicate the high charge transfer resistance of the heterojunction samples, whereas the resistances can be dramatically decreased under light-on illumination due to the high charge mobility and reduced diffusion resistance under light. The open-circuit potential (OCP) can be estimated by the potential values for zero photocurrent, which was analyzed from the pattern of polarization curves. The OCP of the N,S-GQDs/MoSe_2_ heterojunction was approximately −0.09 V vs. RHE, while it was more negative at approximately −0.18 V vs. RHE for the non-doped GQDs/MoSe_2_ heterojunction. The smaller negative value of OCP indicated fewer trapped electrons in the N,S-GQDs/MoSe_2_ heterojunction [38] when compared with GQDs/MoSe_2_. And the N-GQDs/MoSe_2_ was in-between. It is worthwhile that the larger cathodic dark current of the N,S-GQDs/MoSe_2_ heterojunction at −0.20 V vs. RHE, as the benchmark for the onset potential of HER, implying the occurring of HER. Positively sweeping the potential can afford a large enough voltage to boost electrons to the counter electrode. The photocurrents of GQDs/MoSe_2_ or N-GQDs/MoSe_2_ exhibited faster saturation than the N,S-GQDs/MoSe_2_ heterojunction. The electrochemical impedance spectroscopy (EIS) was performed with a frequency that ranged from 0.1 to 10^5^ Hz (Figure 3b), since both 2H and 1T phases coexisted in MoSe_2_, specifically boosting the charge transfer for HER [39]. The semicircle diameters of the Nyquist plots were associated with charge transfer resistance (R_ct_). The lowest R_ct_ of the N,S-GQDs/MoSe_2_ heterojunction implied the highest conductivity, owing to the coexistence of the 2H and 1T phases of MoSe_2_ and the electron donating effects of the N and S dopants. The low R_ct_ implied a high charge transfer ability and long lifetime of the photogenerated electron-hole pairs, offering much improved photocurrent density. The chronoamperometric photocurrents were examined for analyzing the photo-response performance (Figure 3c). Periodic light on/off response was tested at 0.4 V vs. RHE in an electrolyte of 10%(*v*/*v*) lactic acid. Quick photo-responses were observed on anodes with photocurrents rapidly recovered to saturation current in a few seconds. A higher photocurrent of the N,S-GQDs/MoSe_2_ heterojunction was observed compared with the GQDs/MoSe_2_ or N-GQDs/MoSe_2_ heterojunctions. The enhancement of the photocurrent in the heterojunction of the N,S-GQDs/MoSe_2_ is attributed to the fast charge transfer within the system. N and S dopants can enhance the electron density with a larger carrier concentration and better conductivity. Hydrogen evolution of the N,S-GQDs/MoSe_2_ heterojunction is over three times that of the GQDs/MoSe_2_ under simulated sun irradiation (Figure 3d). The hydrogen production rate attained 4.91 μmol h^–1^ cm^–2^ at the apparent quantum efficiency of 7.62% under 420 nm irradiation. N and S dopants can favor the electron-donating and π-delocalization effects of the graphene quantum dots and lead to bandgap contraction, broadening the light absorption spectrum wavelength range. In the UV–visible light absorption spectra (Figure 3e), the light absorption abilities order as N,S-GQDs/MoSe_2_ > N-GQDs/MoSe_2_ > GQDs/MoSe_2_.

The PL spectra in the Figure 3f inset display GQDs, N-GQDs and N,S-GQDs with PL emission centered at 540, 570 and 605 nm, corresponding to the bandgaps of 2.30, 2.18 and 2.05 eV, respectively. N,S dopants introduce more orbitals and further donate and delocalize electrons to facilitate electron transitions together with narrower bandgaps [40,41]. Ultraviolet photoelectron spectroscopy (UPS) was utilized to characterize the Fermi level and work function (Figure 3f). Work function (Φ) was calculated as the difference between the vacuum energy level (E_v_) and the Fermi level (E_F_), i.e., Φ = E_v_ − E_F_. The Φ was determined by the energy difference between the electronic cutoff frequency energy (E_cutoff_) and the excitation energy (21.2 eV). The work function of MoSe_2_ (4.6 eV versus vacuum level) was measured since the excitation energy 21.2 eV minus MoSe_2_ cut-off 16.4 was 4.6 eV, while the work function of N,S-GQD (4.2 eV versus vacuum level) was calculated since the excitation energy 21.2 eV minus N,S-GQD cut-off 16.8 eV was 4.2 eV. The Fermi level (E_f_) was approximately 4.2 eV below the vacuum level. UPS also measured the valence band maximum (VBM), where the VBM with respect to the Fermi level was determined by extrapolating the linear part of the low binding energy region (Figures S1 and S2) where the E_f_-VBM for N,S-GQD and MoSe_2_ is ~1.79 eV and semi-metallic, respectively. The band gaps were also characterized according to UV–vis light absorption and photoluminescence (PL) characterizations. In the (αE)^2^ versus E plot derived from UV–vis (Figure S3), where E denotes photon energy and α denotes the normalized adsorption coefficient, the GQDs, N-GQDs and N,S-GQDs bandgaps can be calculated by the horizontal intercept of the extrapolation from the linear region, verifying the bandgap value (2.05 eV) of N,S-GQDs. Heteroatom doping of the electron-donating N and S species contributed to narrower band gaps, causing stronger and wider light absorption ability, which facilitated a higher capability of hydrogen generation. The band structure and charge transfer mechanisms of the N,S-GQDs/MoSe_2_ heterojunction are illustrated in the Figure 3f inset. The distance between the Fermi level and valance band maximum (VBM) of N,S-GQDs or MoSe_2_ was tested from UPS in Figure S1 or S2 from the Supplementary Material, respectively. Under light, the wavelength smaller than 605 nm (i.e., photon energy larger than bandgap energy 2.05 eV) can be absorbed, separate photogenerated electron-hole pairs and engender the photovoltage. Using a reverse bias can promote a minority of carriers driven toward the counter direction, inducing a photocurrent, although a high energy barrier at the interface did not favor significant photocurrents. The unique dual phases of MoSe_2_ involving semiconductive 2H and metallic 1T could favor the carrier transport through energy-allowable pathways to the electrolyte. The photogenerated electrons rapidly transfer to the metallic 1T MoSe_2_ and induce the hydrogen evolution reaction, while the photogenerated holes transfer via energetically favorable pathway on film, boosting the charge transfer kinetics and inhibiting recombination systematically. Much improved charge separation/transfer kinetics and suppressed recombination of N,S-GQDs/MoSe_2_ in contrast to N-GQDs/MoSe_2_ and GQDs/MoSe_2_ has been evidenced by the photocurrent results and could provide a novel perspective to develop PEC catalysis for the hydrogen evolution reaction and various other applications.

The LSV polarization curve of N,S-GQDs/MoSe_2_ under light irradiation displayed a smaller overpotential of 153 mV at 10 mA/cm^2^, whereas a larger overpotential of 178 mV was displayed without light irradiation (Figure 3g). For comparison, the N-GQDs/MoSe_2_ under light irradiation displayed an overpotential of 196 mV at 10 mA/cm^2^, while rendering an overpotential of 213 mV without light irradiation. In contrast, the GQDs/MoSe_2_ under light irradiation displayed an overpotential of 237 mV at 10 mA/cm^2^, while presenting the largest overpotential of 259 mV in darkness. Light irradiation can significantly boost the HER kinetics; thus, N,S-GQDs/MoSe_2_ under light irradiation possessed the highest activity and current density, owing to the synergistic effect between strong light absorption and improved carrier separation upon heteroatom doping. Tafel slopes of GQDs/MoSe_2_ (79.1 and 75.6 mV dec^−1^), N-GQDs/MoSe_2_ (70.8 and 64.9 mV dec^−1^) and N,S-GQDs/MoSe_2_ (60.4 and 57.3 mV dec^−1^) without light or with light irradiation also demonstrated the similar trend in Figure 3h. Mott–Schottky (M-S) tests were employed to characterize the flat band potentials and semiconductor types (Figure 3i) with the positive slopes near the linear part attributed to the n-type semiconductor. According to the M–S equation,

(1) $$\frac{1}{C^{2}}=\frac{2}{q\varepsilon \varepsilon_{0}N_{d}}(E-E_{fb}-\frac{K_{b}T}{q})$$

where C, q, ε, ε_0_, N_d_, E, K_b_ and T denote the space charge capacitance, electronic charge, dielectric constant of the semiconductor, dielectric constant of permittivity of free space, donor density, potential, Boltzmann’s constant and temperature, respectively. The flat band potential (E_fb_) was calculated from the X intercept via extending the linear fitting part of the MS curves to the X coordinate axis (C^−2^ = 0). The E_fb_ of GQDs/MoSe_2_, N-GQDs/MoSe_2_ and N,S-GQDs/MoSe_2_ was calculated to be −0.43, −0.52 and −0.56 V versus Ag/AgCl, corresponding to 4.27, 4.18 and 4.16 eV below the vacuum level. Moreover, the E_fb_ of the n-type semiconductor was close to the Fermi levels (E_f_). Heteroatom doped N,S-GQDs/MoSe_2_ and N-GQDs/MoSe_2_ possess the E_f_ negative shifts compared to GQDs/MoSe_2_, implying higher photo-induced electron density and stronger reductive capability.

Table S1 shows the comparison of different MoSe_2_ based materials for hydrogen generation reactions. To date, MoSe_2_ is good at photocatalytic or electrocatalytic HER separately [42], but the combination of photocatalytic and electrocatalytic is rare. Thus, it is novel and advantageous to combine both processes for the PEC applications in this work. Morphology characterization and chemical state analysis of the catalysts after PEC reaction experiments were conducted as well. From TEM, HRTEM and SEM observations (Figure S4), the morphology of the N,S-GQDs/MoSe_2_ heterojunction catalyst after the reaction remained similar to that of the N,S-GQDs/MoSe_2_ sample before the reaction in Figure 1a–c, implying its excellent stability. In addition, the XPS characterization data of the N,S-GQDs/MoSe_2_ catalyst after the reaction remained almost unchanged (Figure S5) compared to Figure 2d–i, which also demonstrated the high stability of the chemical states of the N,S-GQDs/MoSe_2_ heterojunction for practical PEC reaction.

#### 2.2.2. Photodegradation Performance

**Figure 4:**
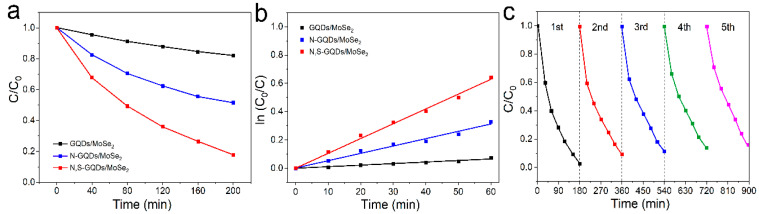
(**a**) Photodegradation efficiencies, (**b**) linear fitting of pseudo first-order kinetics isotherms, and (**c**) cycling runs of photodegradation by N,S-GQDs/MoSe_2_.

For further evidence of the viability of the N,S-GQDs/MoSe_2_ heterojunction, herein we conducted the photocatalytic degradation of RhB in similar conditions and present the results in Figure 4a–c. The RhB concentration slightly decreased using GQDs/MoSe_2_ heterojunction photocatalysis. While the degradation rates obviously increased by using the N,S-GQDs/MoSe_2_ in contrast to the N-GQDs/MoSe_2_ heterojunction, validating more photogenerated carriers generated by N,S-GQDs/MoSe_2_ than either N-GQDs/MoSe_2_ or GQDs/MoSe_2_, owing to stronger light absorption and preferable band gap structure of N,S-GQDs/MoSe_2_. A first-order kinetics equation, ln(C_0_/C) = kt, was applied to determine the kinetic constants (Figure 4b), where the k is approximately 0.0109 min^−1^ for N,S-GQDs/MoSe_2_, which is over 2.1~8.5 times larger than that of N-GQDs/MoSe_2_ (0.0052 min^−1^) or GQDs/MoSe_2_ (0.0013 min^−1^). This phenomenon was attributed to the enhanced photogenerated carriers by N,S-GQDs/MoSe_2_ with an improved bandgap structure and increased light harvesting ability. For recyclable scrutinization and persistency examination (Figure 4c), N,S-GQDs/MoSe_2_ can achieve long-term durability of catalytic activity for over five recycles in the photodegradation of RhB, substantiating its feasible stability and reusability.

## 3. Discussion

In general, the N,S-GQDs/MoSe_2_ heterojunction was prepared via hydrothermal synthesis with the coexistence of metallic 1T phase and semiconductive 2H phase as a result of reductive N_2_H_4_·H_2_O addition. For comparison, the N-GQDs and GQDs were prepared with only N dopants or non-dopants, respectively. The N and S dopants lead to reduced charge transfer barrier, bandgap narrowing, enhanced light absorption and photogenerated charge separation, owing to the electron donating and delocalization effects. The coexistence of 1T and 2H phases of MoSe_2_ also contribute to rapid charge transfer, carrier transport and inhibit photogenerated charge recombination, improving the photocurrents and stability of the N,S-GQDs/MoSe_2_ photoanodes in contrast to the N-GQDs/MoSe_2_ or GQDs/MoSe_2_ heterojunctions. This work offers a new guideline to establish a catalytic heterojunction system, especially surface mediation and bandgap engineering of heterojunctions, for PEC hydrogen evolution reaction and numerous other applications.

## 4. Materials and Methods

N,S-GQDs were hydrothermally synthesized by using citric acid as a carbon source, urea as a N source and thiourea as a S source. Add 1 mmol citric acid, 3 mmol urea and 3 mmol thiourea into 5 mL deionized water, and ultrasonicate for 10 min to dissolve uniformly to form a clear solution. Then transfer the solution to a 20 mL high-pressure autoclave and heat in an oven at 160 °C for 4 h. Then, naturally cool to room temperature to perform continuous dialysis and freeze drying, until the targeted product N,S-GQDs is achieved. In contrast, the N-GQDs were synthesized similarly without using thiourea as the S source, while pristine GQDs were synthesized similarly without using urea or thiourea as the N or S sources, respectively.

Subsequently, heterojunctions between quantum dots and MoSe_2_ were synthesized by another hydrothermal process of two mixed solutions: (1) 4 mmol Se powder dispersed in 10 mL N_2_H_4_·H_2_O and (2) 0.353 g (NH_4_)_6_Mo_7_O_24_·4H_2_O and 5 mg quantum dots dissolved in 20 mL H_2_O. The hydrothermal condition was set at 150 °C for 48 h to convert Mo_7_O_24_^6−^ into Mo^4+^ for the MoSe_2_ generation. The resulting sample was rinsed and centrifugated for 6 times to obtain the target heterojunctions (N,S-GQDs/MoSe_2_, GQDs/MoSe_2_ and N-GQDs/MoSe_2_).

The morphologies of the as-prepared samples were examined by transmission electron microscope (TEM, HT7700, HITACHI, Tokyo, Japan), high-resolution transmission electron microscopy (HRTEM, JEOL JEM-2100F, Tokyo, Japan), aberration-corrected high-angle annular dark-field scanning TEM (AC HAADF-STEM, FEI Talos F200X, Thermo Fisher, Waltham, MA, USA), and field-emission scanning electron microscope (FESEM, Hitachi SU-8010, Tokyo, Japan). The thickness of the as-prepared catalysts was measured by AFM (MultiMode of VEECO, Bruker, Camarillo, CA, USA). The crystallinity structures of the as-prepared heterojunctions were characterized by the X-ray diffraction (XRD) technique (Empyrean 200895, PANalytical B.V., Almelo, The Netherlands) using Cu Kα radiation. The chemistry environments of the as-prepared samples were examined by X-ray photoelectron spectroscopy (XPS) spectra (Escalab 250Xi, Las Condes, Chile) with Al Kα radiation. Surface work function was examined by UV–photoelectron spectroscopy (UPS, AXIS Ultra DLD, Kratos, Manchester, UK) armed with the He-I photon source at hν of 21.2 eV. Raman spectra were measured by WITec (Ulm, Germany) with the model number Alpha 300R and a wavelength laser emitter of 532 nm.

PEC water splitting was operated using a three-electrode system with a heterojunction doped working electrode, saturated calomel electrode as the reference electrode and graphite rod as the counter electrode. The electrolyte of 10% lactic acid was used to examine PEC performance. Light illumination employed simulated AM 1.5 using a Newport xenon lamp. Incident photon-to-electron conversion (IPEC) efficiency measurements were operated by IQE 200TM (Newport) to investigate the photon wavelength-related photocurrent under monochromatic filtered light illumination as monochromator from a Newport 300 W xenon lamp. The H_2_ was examined by gas chromatography (GC, Agilent 7890A, Santa Clara, CA, USA) equipped with a hydrogen flame ionization detector (FID) and thermal conductivity detector (TCD). Heterojunction photoanodes (2 × 2 cm^2^) were applied in the quartz cell with an electrolyte of 130 mL 10% lactic acid. This setup employed the three-electrode system using chronoamperometry technique at the bias of 0.4 V vs. RHE. The conversion between reversible hydrogen electrode (RHE) and Ag/AgCl follows the equation E(RHE) = E(Ag/AgCl) + 0.0591pH + 0.197.

Photo-degradation of the RhB aqueous solution under light irradiation was investigated with a three-electrodes setup containing the as-prepared material-modified paper working electrode; Pt counter electrode and saturated Ag/AgCl reference electrode were put in 20 mL of terephthalic acid solution. After the electrodes were illuminated for different times under magnetic stirring for a specific time period, the solution was centrifuged, and the sample was analyzed with fluorescence emission spectra (at 425 nm) with an excitation wavelength of 315 nm on a F-7100 fluorescence spectrophotometer (HITACHI).

## Data Availability

Data are contained within the article or Supplementary Material.

## Supplementary Materials

The following supporting information can be downloaded at: https://www.mdpi.com/article/10.3390/molecules29051070/s1, Figure S1: Ultraviolet photoelectron spectroscopy (UPS) data of N,S-GQDs.; Figure S2: UPS spectra of MoSe_2_; Figure S3: (a) UV-vis absorption spectra and (b) the (αE)^2^ versus photon energy (E) plots (where α denotes the absorbance coefficient); Figure S4: Morphology characterizations of N,S-GQDs/MoSe_2_ heterojunction catalyst after PEC reaction. (a) TEM image with HRTEM as inset, (b) SEM image of sample nanosheets; Figure S5: (a) Overall XPS, (b) C 1s, (c) Mo 3d, (d) Se 3d, (e) N 1s and (f) S 2p high resolution XPS patterns of the N,S-GQDs/MoSe_2_ heterojunction catalyst after PEC reaction; Table S1: Hydrogen generation comparison of different MoSe_2_-based materials. Refs. [43,44,45] are cited in Supplementary Materials.
