# Supplementary material for: Quantum Dots Mediated Heterojunction Coupling MoSe2 Photoanode for Photoelectrochemical Water Splitting
Source: Molecules. 2024 Feb 29;29(5):1070. doi: 10.3390/molecules29051070 (PMC10935356; doi:10.3390/molecules29051070)
Supplement: Supplementary file 1 [file molecules-29-01070-s001.zip › molecules-2847414-supplementary.pdf]

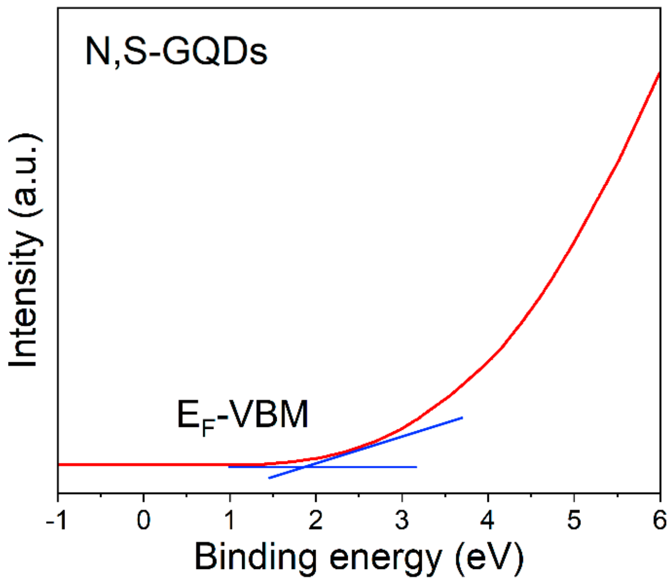

Figure S1. Ultraviolet photoelectron spectroscopy (UPS) data of N,S-GQDs.

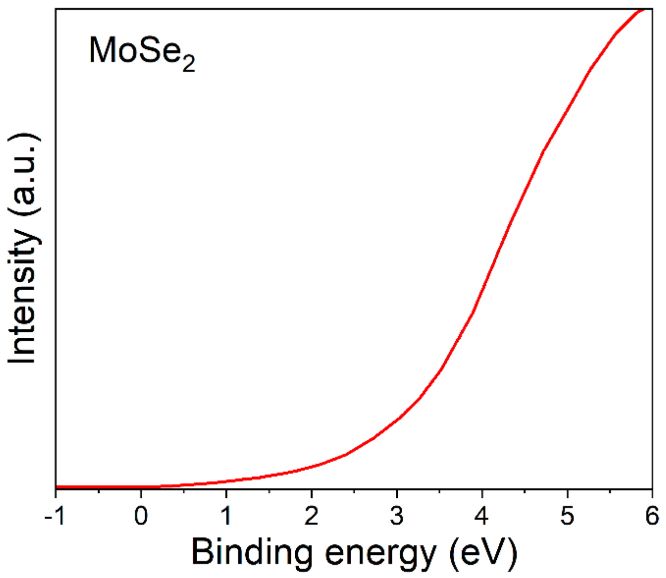

Figure S2. UPS spectra of $MoSe_2$.

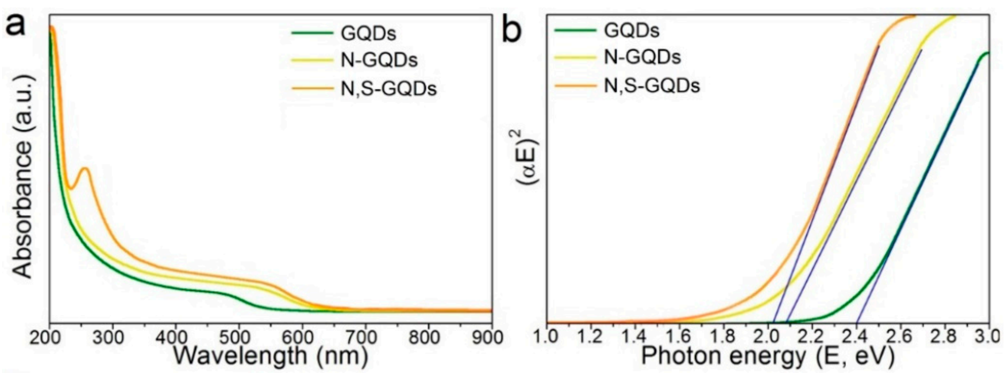

Figure S3. (a) UV-vis absorption spectra and (b) the $(\alpha E)^2$ versus photon energy

(E) plots (where α denotes the absorbance coefficient).

Table S1. Hydrogen generation comparison of different $MoSe_2$-based materials.

Catalysts	Light source	Activity	Overpotential	Tafel	Reference
1T-$MoSe_2$	100 W halogen lamp	62 mmol h^{-1} g^{-1}	——	——	[1]
2H-$MoSe_2$	100 W halogen lamp	0.08 mmol h^{-1} g^{-1}	——	——	[1]
Porous $MoSe_2$	——	——	150 mV at 10 mA cm^{-2}	80 mV dec^{-1}	[2]
$MoSe_2/MoS_2$	——	——	162 mV at 10 mA cm^{-2}	61 mV dec^{-1}	[3]
$MoSe_2/WSe_2$	——	——	275 mV at 10 mA cm^{-2}	80 mV dec^{-1}	[4]
N,S-GQDs/ $MoSe_2$	AM 1.5 Newport xenon lamp	4.91 μmol h^{-1} cm^{-2}	153 mV at 10 mA cm^{-2}	57.3 mV dec^{-1}	This work

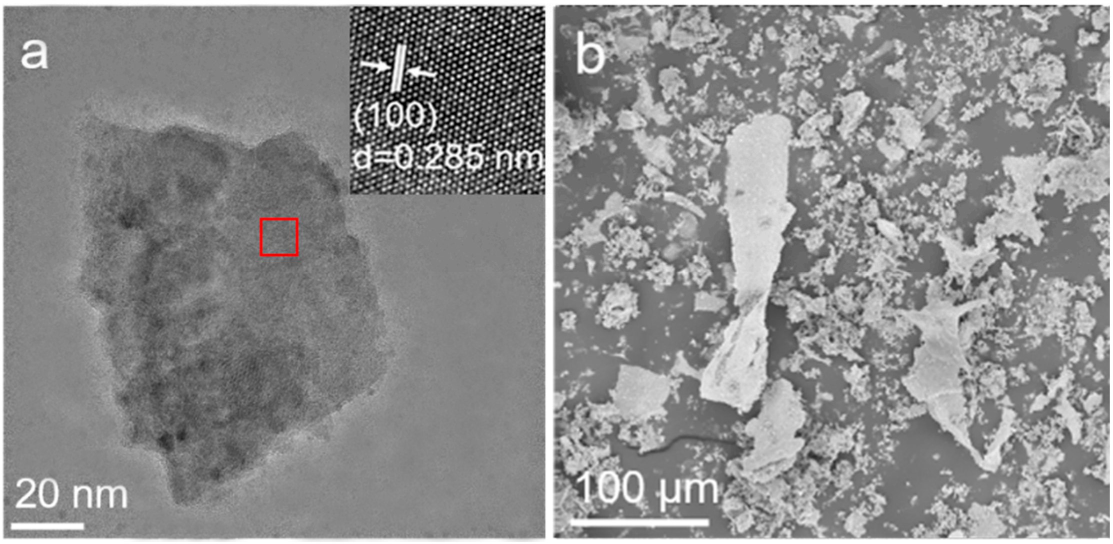

Figure S4. Morphology characterizations of N,S-GQDs/$MoSe_2$ heterojunction catalyst after PEC reaction. (a) TEM image with HRTEM as inset, (b) SEM image of sample nanosheets.

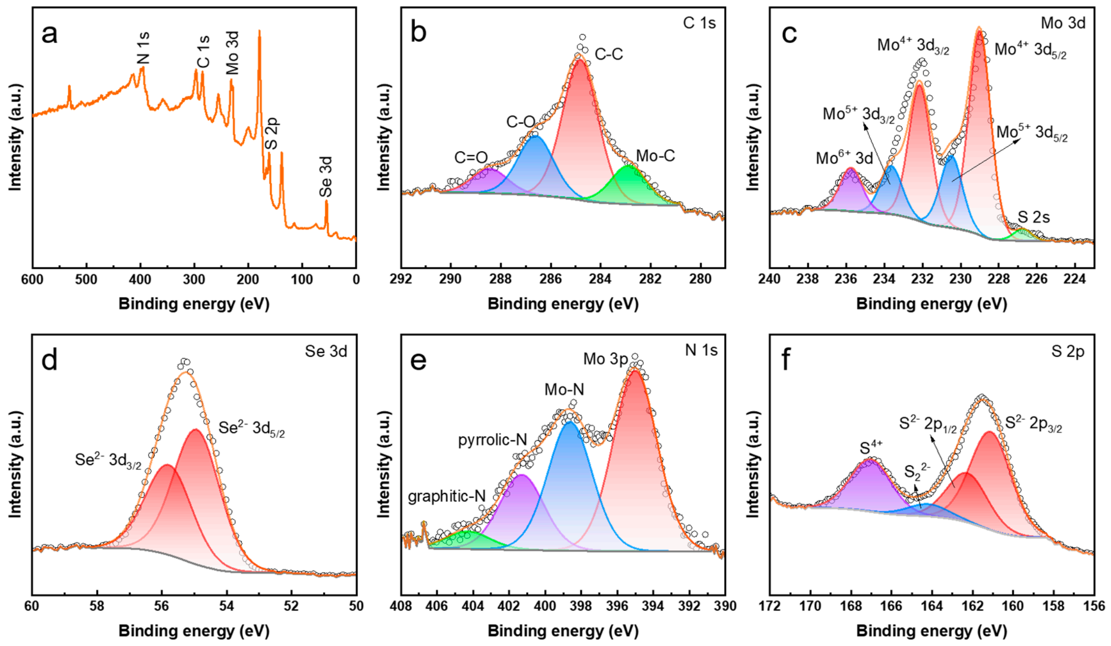

Figure S5. (a) Overall XPS, (b) C 1s, (c) Mo 3d, (d) Se 3d, (e) N 1s and (f) S 2p high resolution XPS patterns of the N,S-GQDs/$MoSe_2$ heterojunction catalyst after PEC reaction.

References

1. Gupta, U.; Naidu, B.; Maitra, U.; Singh, A.; Shirodkar, S.N.; Waghmare, U.V.; Rao, C. Characterization of few-layer 1T-MoSe2 and its superior performance in the visible-light induced hydrogen evolution reaction. *APL Materials* **2014**, *2*, 092802.
2. Lei, Z.; Xu, S.; Wu, P. Ultra-thin and porous MoSe 2 nanosheets: facile preparation and enhanced electrocatalytic activity towards the hydrogen evolution reaction. *Physical Chemistry Chemical Physics* **2016**, *18*, 70-74.
3. Zhao, Y.; Yan, Y.; Lee, J.-M. Recent progress on transition metal diselenides from formation and modification to applications. *Nanoscale* **2022**, *14*, 1075-1095.
4. Singh, H.; Lalla, N.; Deshpande, U.; Arora, S.K. Engineered MoSe2/WSe2 based heterostructures for efficient hydrogen evolution reaction. *Materials Today: Proceedings* **2021**, *45*, 4787-4791.
